# Intelligent quantification of formaldehyde in aquatic product soaking solutions via a novel deep regression framework

**DOI:** 10.3389/fnut.2026.1859033

**Published:** 2026-06-05

**Authors:** Jing-Hao Xi, Ya-Hao Liu, Dan Liu, Ying-Zi Fu, Lun-Zhao Yi, Guang-Hui Fu

**Affiliations:** 1 School of Science, Kunming University of Science and Technology, Kunming, Yunnan, China; 2 Yunnan Key Laboratory of Complex Systemsand Brain-Inspired Intelligence, Kunming University of Science and Technology, Kunming, Yunnan, China; 3 Faculty of Food Science and Engineering, Kunming University of Science and Technology, Kunming, Yunnan, China

**Keywords:** aquatic product soaking solutions, deep regression, formaldehyde, image-to-value mapping, multi-scale feature aggregation

## Abstract

**Introduction:**

The illicit use of formaldehyde (FA) as a preservative in aquatic products poses grave public health risks due to its proven carcinogenicity. While gold-standard techniques like HPLC offer high precision, their implementation in real-time surveillance is hindered by labor-intensive pre-treatment and reliance on expensive instrumentation, necessitating intelligent, non-destructive sensing platforms.

**Methods:**

We present Res-Reg, a novel image-to-value deep regression framework optimized for rapid FA quantification. The sensing paradigm integrates the high specificity of the Hantzsch chromogenic reaction with digital microscopic imaging, employing a residual-based backbone and a specialized regression head for multi-layer feature aggregation. To enhance environmental robustness against matrix interference, the training dataset was augmented with Gaussian blur kernels.

**Results:**

Systematic evaluation on 1,548 microscopic images demonstrated that Res-Reg achieved an MAE of 0.0027, MSE of 0.0028, and GM of 0.0002, significantly outperforming traditional CNN-based models by maintaining superior sensitivity in low-concentration intervals where optical signals are often obscured by turbidity.

**Discussion:**

Res-Reg effectively bridges the gap between microscopic optical signatures and precise chemical concentration without complex purification. This modular and scalable technology supports the digital transformation of food quality control, providing a potent solution for proactive safety surveillance and the preservation of global nutritional standards in the Food Industry 4.0 era.

## Graphical Abstract

**Figure:**
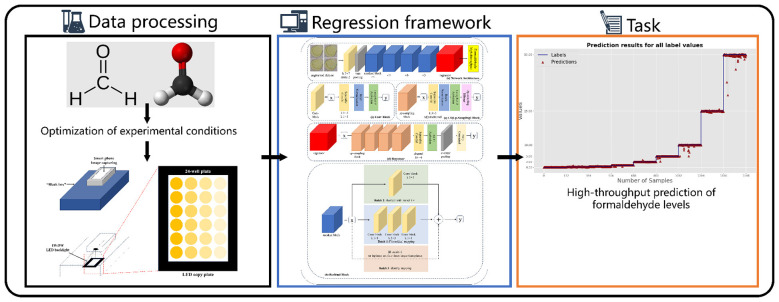

## 1 Introduction

In the contemporary globalized food market, ensuring nutritional integrity and safeguarding consumers against chemical adulteration remains a paramount challenge for public health authorities. Formaldehyde (FA), a highly reactive and volatile aldehyde, has gained notoriety as both an environmental pollutant and a pervasive illicit additive. Despite stringent regulatory bans, FA is frequently employed by unscrupulous producers to extend the shelf-life and enhance the textural appeal of perishable high-protein matrices, such as aquaculture products, soy-based derivatives, and various processed meats (1). The toxicological profile of FA is devastating; the International Agency for Research on Cancer (IARC) has definitively classified it as a Group 1 human carcinogen. Beyond its systemic carcinogenic potential, recent nutritional studies suggest that even trace-level FA ingestion can significantly alter the bioaccessibility of essential amino acids and disrupt gut microbiota homeostasis, leading to compromised nutrient metabolism (2). Consequently, the transition from reactive regulation to proactive, high-throughput screening of FA is essential for maintaining the nutritional safety of the global food supply chain.

The quantification of FA in complex food matrices traditionally relies on sophisticated instrumental paradigms, including High-Performance Liquid Chromatography (HPLC) and Gas Chromatography-Mass Spectrometry (GC-MS) (3). Among chromogenic strategies, the Hantzsch reaction remains the bedrock of FA analysis due to its exceptional stoichiometry and specificity (4). In this reaction, FA undergoes a cyclization with acetylacetone and ammonium acetate to synthesize 3,5-diacetyl-1,4-dihydrolutidine (DDL), a stable yellow chromophore that can be quantified via spectrophotometry (5). While these methods offer peerless sensitivity, their integration into real-time food monitoring is hindered by the hegemony of “centralized laboratory” logistics. These constraints include labor-intensive sample purification, the requirement for toxic organic solvents, and a lack of portability for onsite inspection at markets or production facilities (6, 7). Recent innovations in Paper-based Analytical Devices (PADs) and Microfluidic PADs (μPADs) have attempted to decentralize this process (8), offering low-cost, disposable platforms for colorimetric assays (9). However, naked-eye interpretation of these sensors is inherently subjective and prone to “false-negative” results, particularly when the analyte concentration is near the limit of detection (LOD) or when background interference from the food matrix obscures the color transition (10).

The emergence of Food Industry 4.0 (11, 12) has catalyzed the integration of smartphone-based sensing with artificial intelligence (AI) to provide objective, non-destructive analytical solutions (13). Early iterations of intelligent sensing primarily utilized traditional machine learning (ML) classifiers–such as Support Vector Machines (SVM), Random Forest (RF), and Artificial Neural Networks (ANN) (14)—to map colorimetric shifts across various color spaces, including RGB, HSV, and CIE Lab* (15). These models have shown promise in quantifying glucose in saliva and identifying flavor profiles in distilled spirits. Nevertheless, traditional ML algorithms often demonstrate a “performance ceiling” when faced with the non-linear optical noise common in field settings, such as fluctuating ambient lighting, varying camera sensitivities, and focus blur (16–18). While Deep Learning (DL) architectures like Convolutional Neural Networks (CNNs) have revolutionized image-based food quality assessment (19, 20), a significant methodological gap remains in continuous chemical regression (21, 22). Most extant DL models are optimized for discrete classification (23); when repurposed for chemical quantification, they often lack the fine-grained sensitivity required to distinguish subtle concentration variations in the "gray zones" of food contamination.

To bridge the gap between microscopic chemical sensing and precise numerical estimation, this study proposes Res-Reg, a novel deep-regression architecture designed for the high-throughput quantification of formaldehyde. Our approach synergizes the rigorous chemical kinetics of the Hantzsch reaction with a deep residual backbone optimized for feature aggregation. Unlike conventional systems that rely on macroscopic smartphone photography, the Res-Reg system utilizes microscopic imaging to capture the distribution of reaction products at a granular level, effectively amplifying the signal-to-noise ratio in low-concentration samples. To enhance environmental robustness, we incorporated a data-augmentation pipeline using Gaussian blur kernels (24), simulating the optical degradations common in onsite sensing (25). By reconstructing the traditional CNN output layer into a specialized regression head, Res-Reg facilitates a direct, ultra-accurate “image-to-value” mapping that bypasses the resolution limits of both human vision and traditional classification-based AI.

The contributions of this research to the field of nutritional science and food safety are threefold:

1. Holistic Chemical-Digital Integration: We report an end-to-end detection ecosystem that harmonizes optimized chemical stoichiometry (e.g., reagent concentration and incubation kinetics) with digital sensing, ensuring the acquisition of high-fidelity data across a diverse concentration spectrum.
2. Precision-Engineered Regression Architecture: We introduce a multi-scale feature aggregation mechanism within the Res-Reg framework. This design significantly enhances the model's ability to perceive infinitesimal optical shifts, achieving laboratory-grade precision in formaldehyde detection without the need for centralized instrumentation.
3. Versatility and Onsite Scalability: The Res-Reg system eliminates the bottleneck of complex sample pretreatment, allowing for rapid, automated safety assessments in dynamic industrial environments. The modular architecture of the regression head provides a versatile blueprint for the intelligent monitoring of other critical food contaminants or nutritional markers in diverse food matrices.

## 2 Materials and methods

### 2.1 Materials

A suite of high-precision analytical instruments and mobile sensing devices was employed for data acquisition and sample processing. To replicate authentic food safety monitoring scenarios, formaldehyde-spiked aquatic product soaking solutions were prepared as simulated food samples, accounting for the complex biochemical background and turbidity inherent in seafood matrices. The absorbance profiles and ground-truth concentrations of these simulated samples were determined using a dual-beam UV-Vis spectrophotometer (Beijing Purui General Instrument Co., Ltd.). Sample homogenization and the elimination of micro-bubbles were facilitated by a KS-500DB LCD ultrasonic cleaner (Kunshan Jielimei Ultrasonic Instrument Co., Ltd.). All experimental solutions were prepared using ultra-pure Type I water (Nanjing QuanKun Biotechnology Co., Ltd.) in strict compliance with the GB/T6682-2008 standard.

For the digital sensing and image acquisition phase, a heterogeneous smartphone array–comprising the iPhone 11, iPhone 14, OPPO Reno5, and Redmi Note 11t Pro–was utilized to capture the chromogenic response. This multi-device approach was designed to adapt to the application scenario of migrating to mobile devices and validate the robustness of the Res-Reg framework against varying sensor sensitivities and hardware architectures. All chemical reagents used for the chromogenic Hantzsch reaction were of analytical grade, including formaldehyde standard solution (10 mg/ml), acetylacetone, and ammonium acetate (Shanghai McLean Biochemical Technology Co., Ltd.), alongside glacial acetic acid (Tianjin Fengchuan Chemical Reagent Technology Co., Ltd.).

### 2.2 Optimization of experimental conditions

The aim of this experiment is to achieve rapid detection of formaldehyde through the optimization of experimental conditions. The chemical basis of the colorimetric shift is rooted in the Hantzsch reaction: formaldehyde (FA) reacts with acetylacetone in the presence of ammonium acetate to form a yellow-colored product, 3,5-diacetyl-1,4-dihydrolutidine (DDL). This lutidine derivative exhibits a characteristic absorption peak at approximately 415 nm. As the FA concentration increases, the intensity of this yellow chromophore strengthens, providing the optical foundation for the Res-Reg framework to perform quantitative regression based on pixel-level chromatic variations. The specific optimized conditions include parameters such as the amount of acetylacetone added, reaction time, and reaction temperature. The specific optimization steps are as follows.

The experiment first optimizes the effect of acetylacetone solution on formaldehyde detection by adding different volumes of acetylacetone solution to a standard solution. The specific procedure is as follows: 2 mL of 25 mg/L formaldehyde solution is transferred into a 10 mL colorimetric tube. Different volumes of 4.0 g/L acetylacetone solution (1, 2, 3, 4, and 5 mL) are added, and the volume is adjusted to the calibration mark with aquatic product soaking solution, followed by thorough mixing. All reaction mixtures are placed in a 70°C water bath for 15 minutes, then cooled to room temperature. The absorbance is measured at a wavelength of 415 nm, and the experiment was repeated six times.

**Figure 1:**
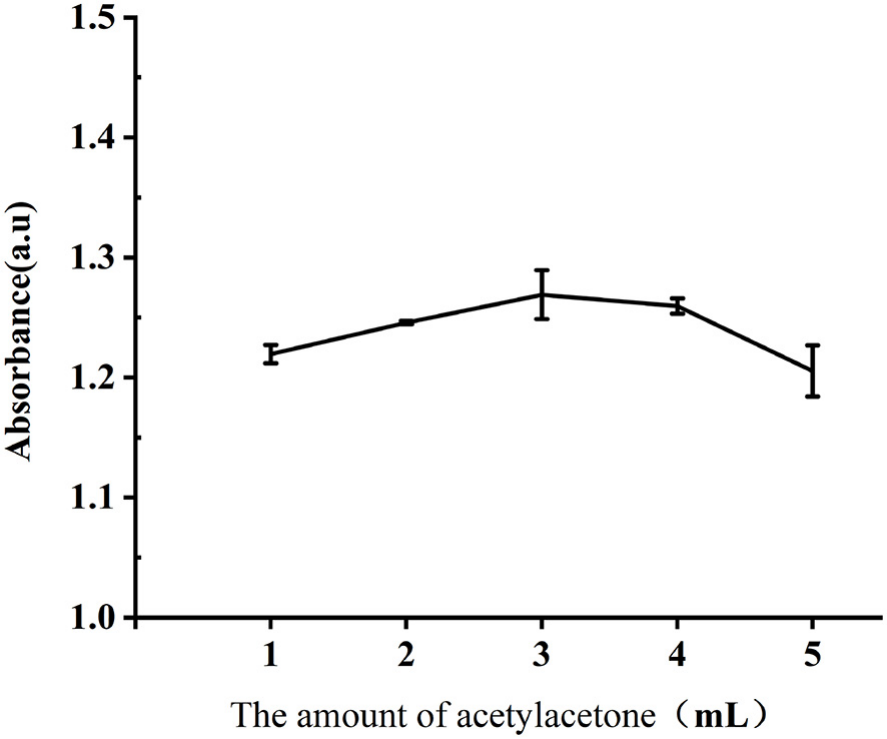
Effect of acetylacetone dosage (mL) on absorbance at 415nm.

The optimization results indicate that the amount of acetylacetone added has a significant impact on the absorbance value (as shown in Figure 1). As the amount of acetylacetone increases, the absorbance shows a gradual upward trend, reaching a maximum value at an addition of 3 mL, after which it levels off. Therefore, the appropriate addition of acetylacetone is crucial for enhancing the reaction sensitivity for formaldehyde detection.

The choice of reaction time and temperature has a significant impact on the efficiency of the reaction between formaldehyde and acetylacetone. To optimize the reaction conditions, the experiment was conducted at different temperatures (50°C, 60°C, 70°C, 80°C) and various reaction times (5 min, 10 min, 15 min, 20 min, 25 min). After adding 3 mL of acetylacetone solution, the mixture was reacted under each condition, then cooled to room temperature. The absorbance was immediately measured at 415 nm, and the experiment was repeated six times.

**Figure 2:**
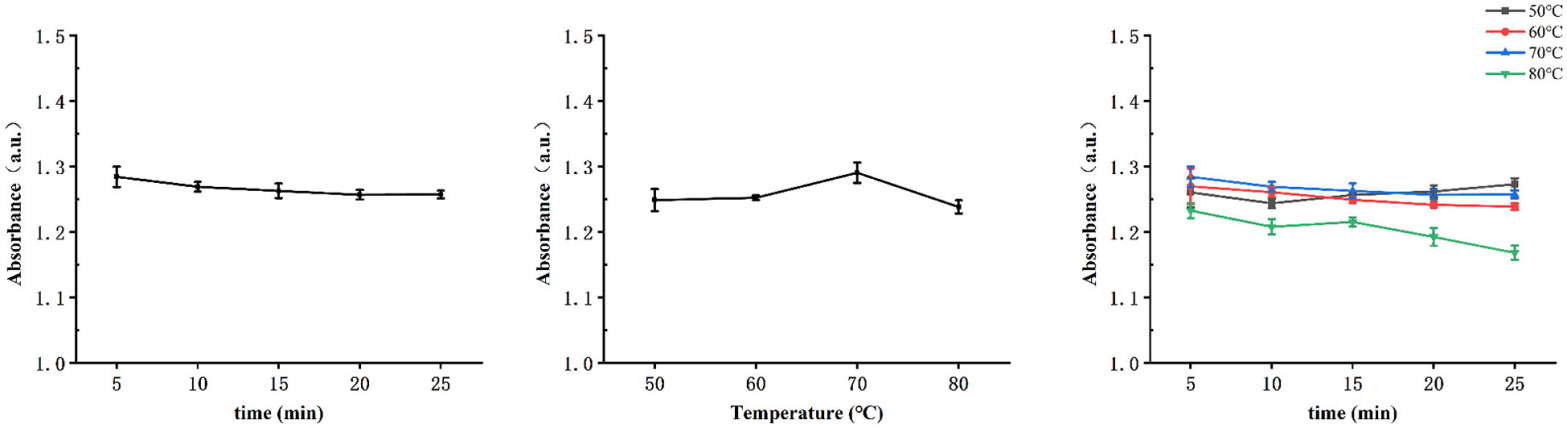
Optimization of reaction kinetics: Effects of temperature and duration on the absorbance of the Hantzsch reaction product.

The results indicate that both reaction temperature and reaction time have a significant impact on absorbance (as shown in Figure 2). When the reaction temperature was 70°C and the reaction time was 15 minutes, the absorbance reached its optimal value, indicating that the efficiency and stability of the reaction were most favorable at these conditions. Therefore, reaction conditions of 70°C and 15 minutes were selected as the optimal experimental.

### 2.3 Imaging process

Based on the conclusions of the experimental conditions optimization, the 2 mL of a 25 mg/L formaldehyde standard solution was added to a 10 mL colorimetric tube, followed by the addition of 3 mL of acetylacetone. The mixture was then heated in a 70°C water bath for 15 minutes to facilitate the reaction. After the reaction, 1 mL of the reaction mixture was transferred to a single well of a 24-well plate for subsequent image acquisition and analysis. The color of the resulting solution varied with formaldehyde concentration, and this phenomenon was utilized for quantitative concentration analysis.

**Figure 3:**
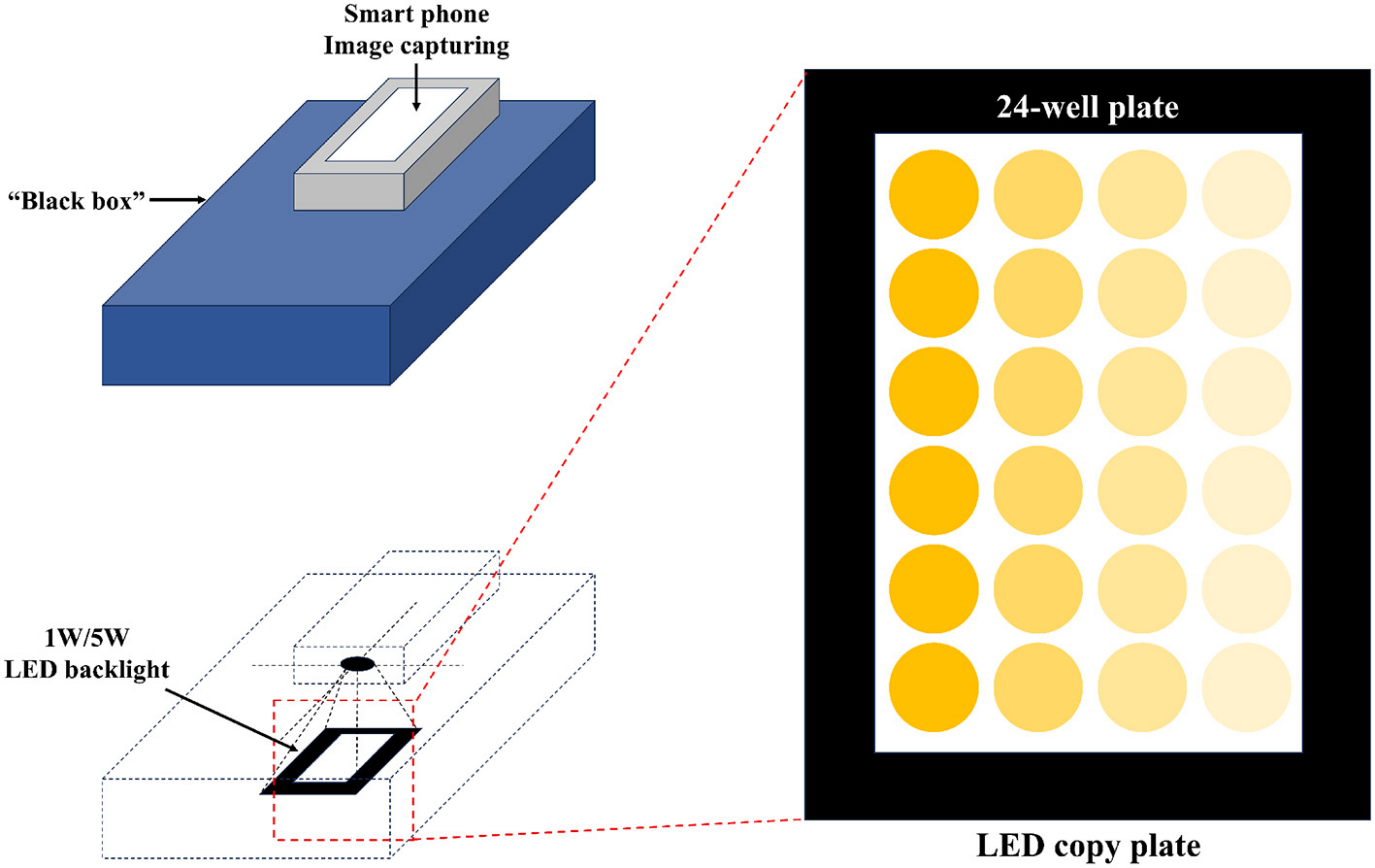
Schematic representation of the customized image acquisition device for smartphone-based digital sensing.

An image acquisition device specifically designed for smartphone photography was developed for the experiment, as shown in the schematic diagram (Figure 3). First, an opaque black cardboard was placed over an LED backlight panel with power outputs of 1W and 5W. The power levels (1W and 5W) were regulated via a constant current driver with a physical toggle switch to ensure stable luminance throughout the acquisition process. According to the manufacturer's specifications, the LED employs a blue-chip-on-phosphor architecture, yielding a typical white-light spectrum with a primary emission peak at approximately 450 nm and a broad secondary peak between 540–600 nm. This consistent wavelength distribution ensures that the primary variations between the two modes are related to photon flux (intensity) rather than significant spectral shifts, thereby providing a controlled environment for testing the model's adaptive capacity. A region matching the size of the 24-well plate was cut out in the center of the cardboard to ensure that the 24-well plate received uniform white background lighting. To prevent interference from external light, a 200-mm-high black box was used to cover the device. The smartphone captured images of the sample through an opening at the top of the box. To ensure image consistency and avoid edge shadows, a circular region with a diameter of 200 pixels, centered on the reaction well, was selected for analysis.

**Figure 4:**
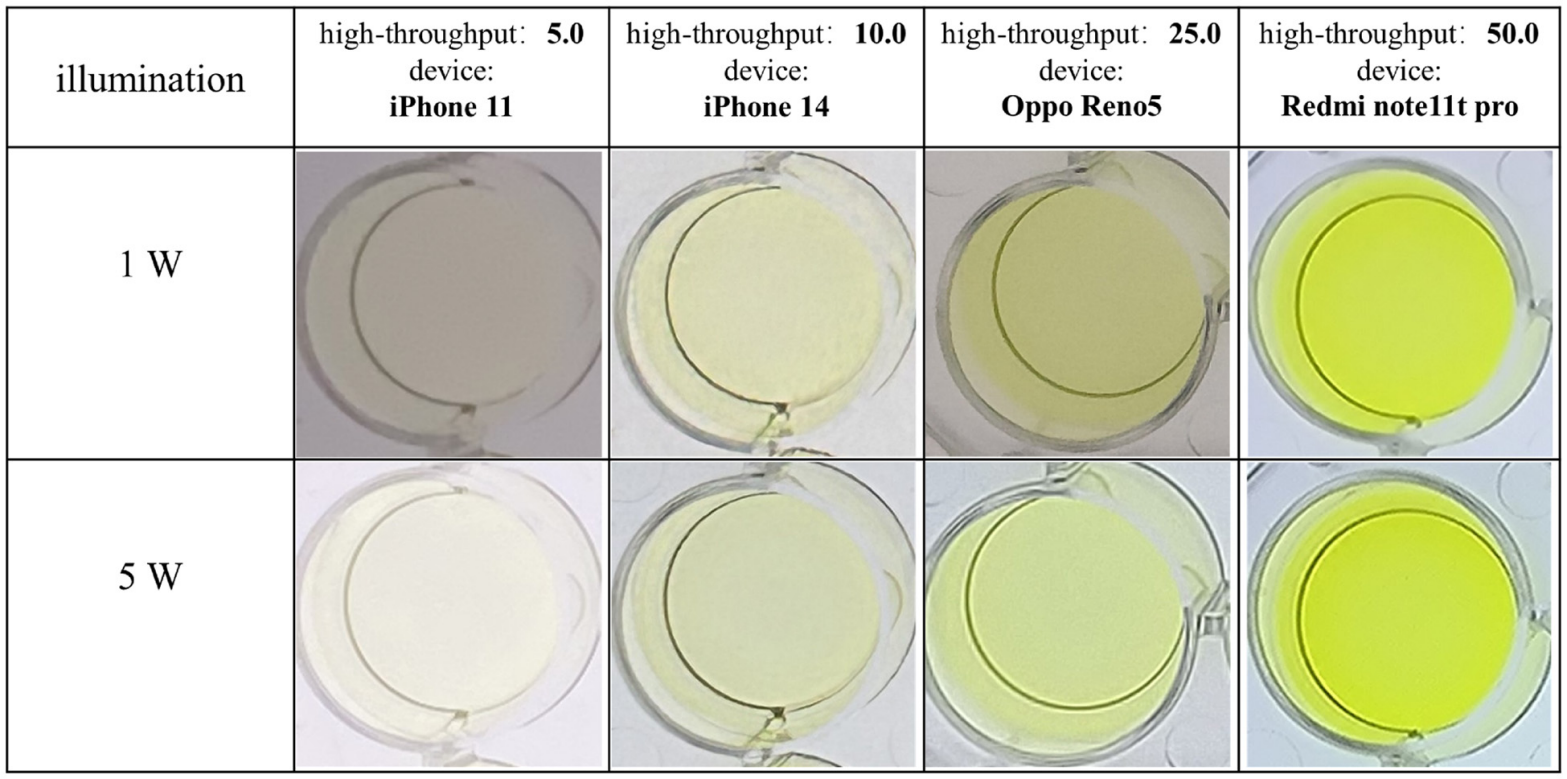
Representative images of formaldehyde samples captured via four different mobile devices under 1W and 5W illumination.

To validate the cross-platform robustness of the Res-Reg framework, four smartphones with diverse hardware architectures were employed: iPhone 11, iPhone 14, OPPO Reno5, and Redmi Note 11t Pro. These devices feature significant variations in imaging hardware, with CMOS sensor resolutions ranging from 12 MP to 64 MP and apertures spanning from f/1.5 to f/1.9. Due to disparities in sensor size, pixel pitch, and proprietary Image Signal Processor (ISP) algorithms, each device exhibits distinct characteristics in terms of light sensitivity, dynamic range, and noise profiles (as shown in Figure 4). This hardware heterogeneity intentionally introduces optical variance, allowing for a rigorous evaluation of the model's ability to maintain predictive consistency across varying levels of image quality.

**Figure 5:**
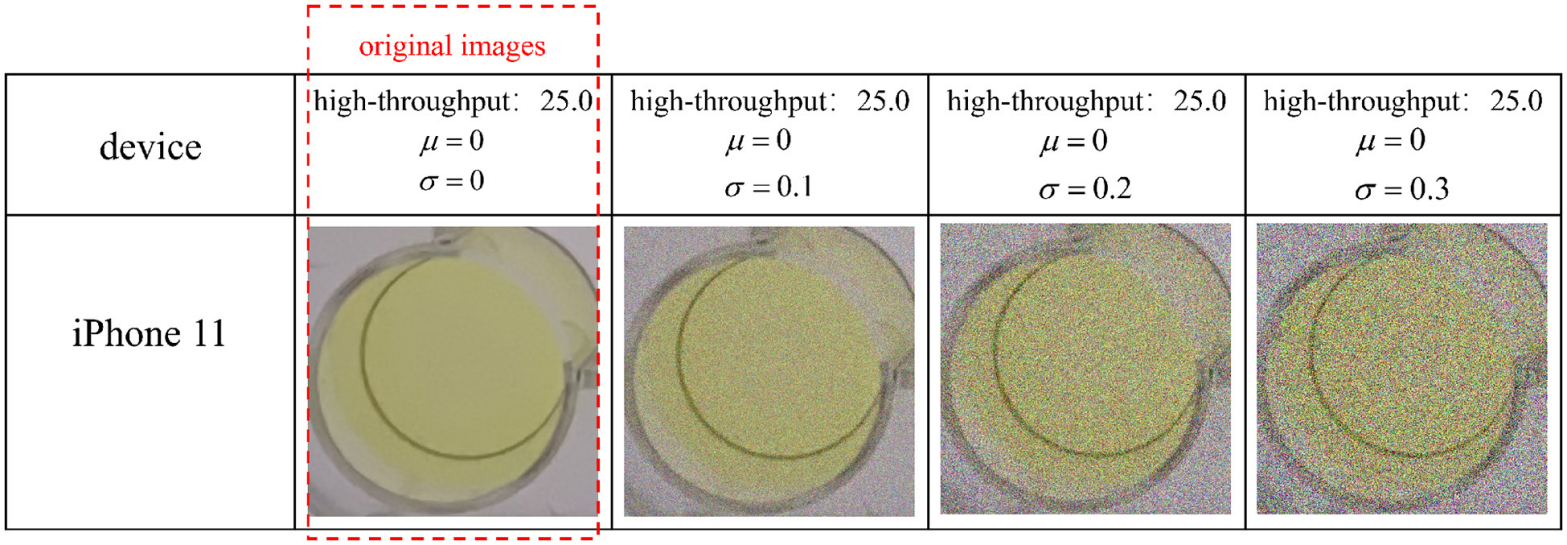
Examples of data augmentation using Gaussian blur with varying kernels (σ = 0 to 0.3) to simulate environmental noise.

A total of 2,593 microscopic images were collected for this experiment, with all images sourced from four common mobile devices. To ensure diversity in the experiment, the images were captured under two different light source power settings (1W and 5W), as shown in Figure 4. Given the limitations in acquiring labels for the dataset, Gaussian blurring was employed to augment the dataset. Specifically, four different blur parameters (0, 0.1, 0.2, 0.3) were applied to the original images, resulting in an expanded dataset that quadrupled in size, from the initial 2,593 image samples to 10,372, as illustrated in Figure 5.

### 2.4 Data augmentation employing Gaussian blur

The dataset used for training is often limited by the difficulty of acquiring large amounts of image data. To address this, Gaussian blur (26, 27) is applied as a data augmentation method to expand the limited dataset. Gaussian blur is an image-blurring technique that simulates the effect of viewing an image through a translucent screen. This approach not only augments the dataset by generating additional samples based on limited data sources but also simulates the image acquisition quality under suboptimal sampling conditions. Gaussian blur is a smoothing process achieved through convolution, where the blurring operation is based on the convolution of the image with a Gaussian function. Given a pixel *f*(*x, y*) in the image and a Gaussian kernel *G*_σ_(*x, y*), the blurred image *g*(*x, y*) can be represented as Equation 1:


$$\begin{array}{l} g\left(x,y\right)=\left(f*G_{\sigma }\right)\left(x,y\right), \end{array}$$ (1)

where (f*G_σ_) denotes the convolution operation between *f*(*x, y*) and *G*_σ_(*x, y*). Alternatively, it can be represented as Equation 2:


$$\begin{array}{l} g\left(x,y\right)=\int \int f\left(x-u,y-v\right)G_{\sigma }\left(u,v\right)dudv, \end{array}$$ (2)

where the Gaussian kernel *G*_σ_(*x, y*) is defined as Equation 3:


$$\begin{array}{l} G_{\sigma }\left(x,y\right)=\frac{1}{2\pi \sigma^{2}}e^{-\frac{x^{2}+y^{2}}{2\sigma^{2}}}, \end{array}$$ (3)

where σ represents the contribution of the central pixel to the calculation result. A smaller σ value means that the blur effect is more dominated by the central pixel, resulting in a lower degree of blurring. Conversely, a larger σ increases the degree of blurring, making the image appear significantly more blurred (26).

### 2.5 Res-Reg framework

**Figure 6:**
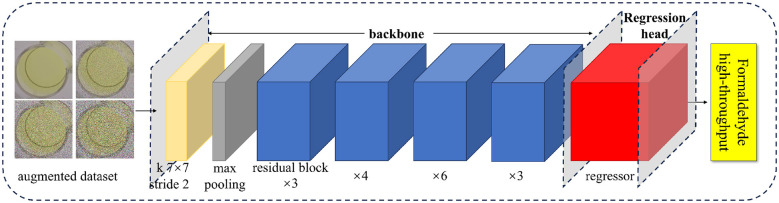
Detailed architecture of the proposed Res-Reg deep regression framework, including the feature backbone and specialized regression head.

This study proposes a regression architecture, Res-Reg, for high-throughput formaldehyde estimation in food. The specific details of the architecture are illustrated in Figure 6. The regression architecture consists of a backbone network and a regression head. Initially, the image dataset is expanded to four times the size of the original dataset through data augmentation, which enhances the diversity and robustness of the dataset. Next, the backbone network extracts key features from the expanded dataset. Finally, the regression head processes the output from the backbone network to achieve high-throughput formaldehyde estimation.

#### 2.5.1 Backbone

**Figure 7:**
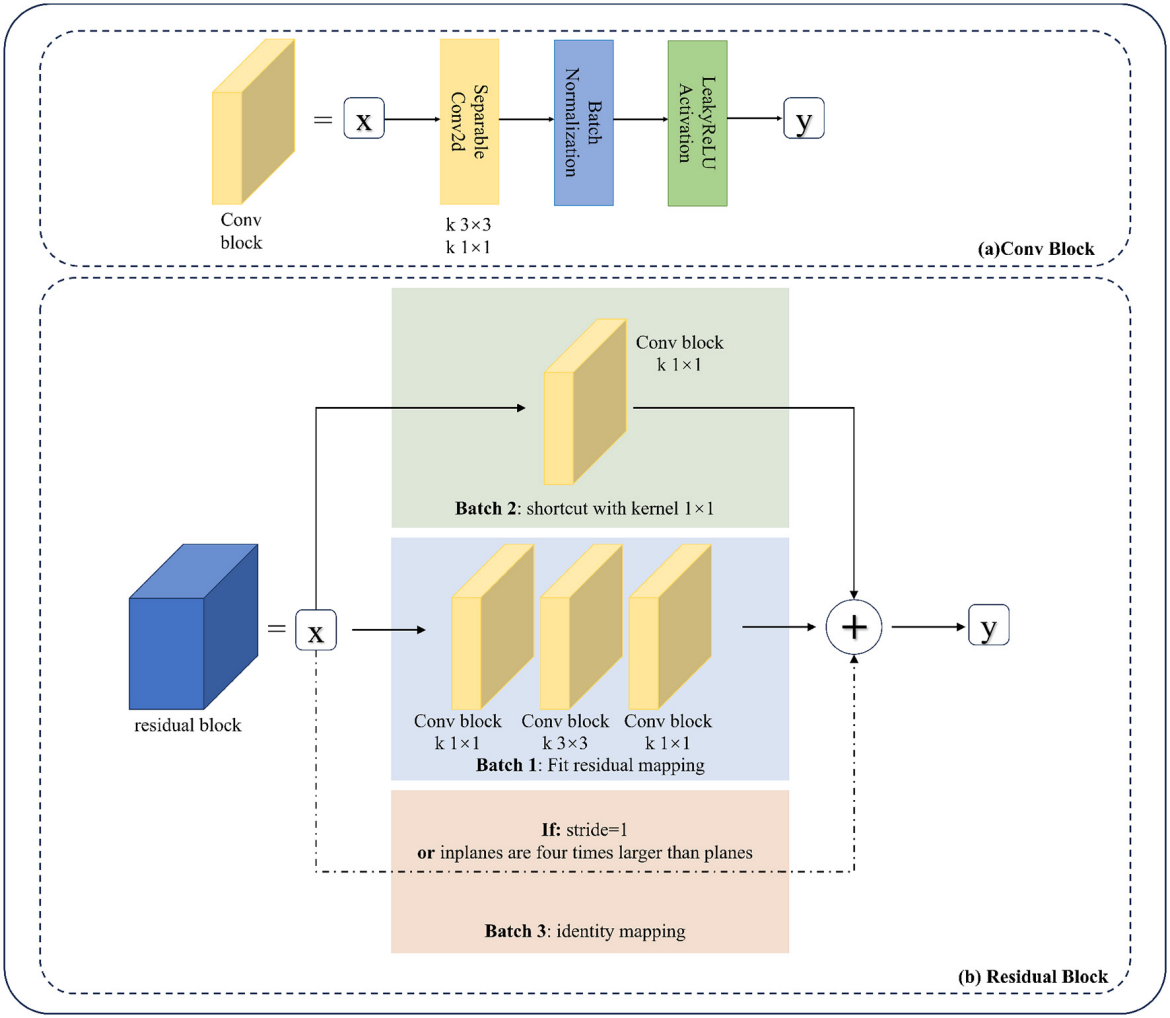
Internal structure of the Res-Reg backbone modules: **(a)** Depth-wise separable convolution block; **(b)** Multi-branch residual block.

Since its introduction in the ImageNet competition in 2015, residual neural networks (ResNet) (28) have been widely applied due to their practicality. As shown in Figure 6, the backbone network is responsible for computing the convolutional feature maps of the entire input image and primarily consists of multiple multi-branch residual block modules (as shown in Figure 7b). Each stacked multi-branch residual block is composed of several convolutional blocks (as shown in Figure 7a), where each convolutional block consists of convolutional layers, batch normalization layers, and activation functions.

The activation function used is LeakyReLU (Leaky Rectified Linear Unit) for activation, where the neuron input is defined as *x*. The definition of LeakyReLU is equation (Equation 4):


$$f\left(x\right)=\{\begin{array}{ll} x, & x\geq 0\\ negative\_slope\cdot x, & x<0 \end{array}$$ (4)

The default value of *negative*_*slope* is 0.01, which is typically a small positive floating-point number used to control the slope of the linear part in the negative input range. LeakyReLU is an improved version of the Rectified Linear Unit (ReLU), which maintains linearity in the non-negative input range and introduces a small slope in the negative range to prevent the vanishing gradient problem. This design allows LeakyReLU to retain the advantages of the traditional ReLU activation function while effectively mitigating the gradient issues caused by negative inputs, thereby enhancing the stability of the model training process.

Each module in the backbone network contains a varying number of multi-branch residual blocks, with each residual block consisting of three branches. Branch 1 consists of three convolutional blocks (as shown in Figure 7a) followed by a LeakyReLU activation function, which is responsible for approximating the residual mapping. Branch 2 features a shortcut connection with a convolutional block of kernel size 1 × 1. Branch 3 represents the identity mapping, which is performed when the stride is 1 or the number of input channels is four times the number of output channels. Specifically, Branch 1 includes depth-wise spatial convolutions performed on each input channel, followed by pointwise convolutions to adjust the number of channels. Depth-wise spatial convolutions are used to extract features from each input channel, while pointwise convolutions significantly reduce the computational and parameter complexity. A LeakyReLU activation function is added at the end of Branch 1 to transform the input signal into a differentiable operation for output, effectively preventing the “dying neuron” (29) problem (i.e., some neurons may never be activated during training). Additionally, each residual block incorporates a residual structure, including the convolutional block in Branch 2 and the identity mapping in Branch 3, to prevent the vanishing gradient problem and facilitate information flow. This structure ensures that the network can maintain effective information flow even as its depth increases during training.

#### 2.5.2 Regression head

**Figure 8:**
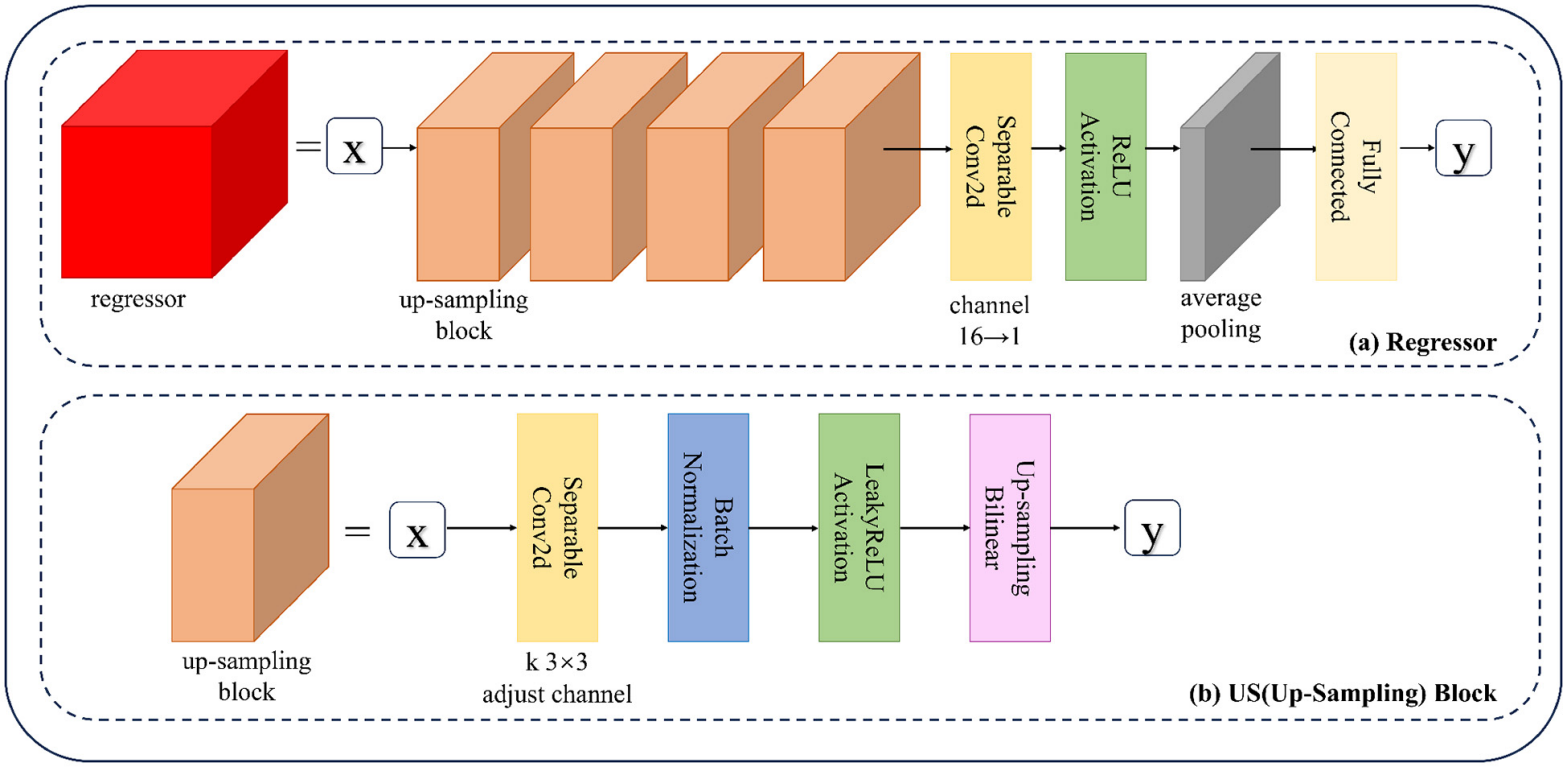
Components of the regression head: **(a)** Global information regressor; **(b)** Multi-scale upsampling (US) block using bilinear interpolation.

To effectively obtain prediction results from the feature maps trained by the backbone network, a regressor is constructed (as shown in Figure 8a) to aggregate the information. Through a series of upsampling (US) blocks (as shown in Figure 8b), the feature map's size and features are gradually enlarged, ultimately producing a density map with a single channel. Finally, the information is aggregated via an average pooling layer and output through a fully connected layer to provide the estimated value. This design effectively integrates features from different scales, ensuring efficient feature extraction while enabling the regressor to accurately predict formaldehyde content. The use of upsampling blocks further enriches feature representation, enhancing the model's adaptability to the high-throughput estimation task.

The US blocks consist of convolutional layers, LeakyReLU activation functions, and upsampling layers. The convolutional layers are used to extract features and gradually adjust the number of channels as the data passes through the module. The upsampling layer employs bilinear interpolation to increase the spatial resolution of the feature map, providing appropriately sized inputs for the subsequent network layers. After passing through four US blocks, the feature map undergoes pointwise convolution using a 1 × 1 convolutional layer to generate a density map with a single channel. Since the regression targets are all positive, a ReLU activation function (30) is applied after the convolutional layer (as shown in Equation 5) to map any real number to a non-negative value. This structural design effectively enhances the spatial details of the feature map while ensuring that the output estimates comply with the requirements of the regression task through the use of the ReLU activation function. The combination of upsampling and pointwise convolution ensures the model performs well and accurately when handling high-resolution density maps. The definition of ReLU is as follows:


$$\begin{array}{l} \text{Re}LU\left(x\right)=\max\left(0,x\right) \end{array}$$ (5)

To prevent overfitting and reduce the number of parameters, an average pooling layer is added after the US blocks. Subsequently, the regressor outputs a floating-point value as the final estimate through a fully connected layer. This design not only effectively reduces the complexity of the model, but also smooths the feature map through the average pooling layer, aiding the model in generalizing better and ultimately ensuring efficient and accurate estimation results.

#### 2.5.3 Visualizing Res-Reg by Grad-CAM

To achieve a visual interpretation of our Res-Reg regression framework, we utilized the Gradient-weighted Class Activation Mapping (Grad-CAM) (31) methodology. Traditionally employed in classification tasks, Grad-CAM leverages the gradient information flowing into the last convolutional layer of a CNN to generate localization maps, highlighting regions most relevant to a specific target class *c*. In this standard formulation, the importance weight $\alpha_{c}^{k}$ for a feature map *A*^*k*^ is calculated via global average pooling of the gradients of the class score *y*_*c*_ (before softmax):

where the importance weight is defined as Equation 6:


$$\begin{array}{l} \alpha_{c}^{k}=\frac{1}{Z}\underset{i}{\sum }\underset{j}{\sum }\frac{\partial y_{c}}{\partial A_{ij}^{k}}, \end{array}$$ (6)

where *Z* = *u*×*v* is the total number of spatial locations in the feature map, and $A_{ij}^{k}$ represents the activation at spatial location (*i, j*). The weights are then used to perform a weighted combination, followed by a ReLU activation to yield the categorical heatmap:

which is defined as Equation 7:


$$\begin{array}{l} L_{c}^{Grad-CAM}=ReLU\left(\underset{k}{\sum }\alpha_{c}^{k}A^{k}\right) \end{array}$$ (7)

Unlike traditional classification models that predict discrete categories, our Res-Reg framework outputs a continuous scalar value. Therefore, we adapted Grad-CAM for the regression task by replacing the categorical class score *y*_*c*_ with the continuous regression output *y*_*r*_, which represents the predicted formaldehyde concentration.

Crucially, this adaptation shifts the physical meaning of the visualization. In this regression context, the modified importance weight $\alpha_{r}^{k}$ mathematically reflects the sensitivity of the predicted concentration to the activations in the *k*-th feature map. By computing the partial derivative $\frac{\partial y_{r}}{\partial A_{ij}^{k}}$, we can precisely identify the spatial regions that positively contribute to a higher concentration estimate. Consequently, the resulting heatmap functions as visual evidence of the “chromogenic signal intensity” (i.e., the localized accumulation of the 3,5-diacetyl-1,4-dihydrolutidine chromophore) perceived by the model, rather than a mere categorical probability. The regression-adapted localization map is defined as Equation 8:


$$\begin{array}{l} L_{r}^{Grad-CAM}=ReLU\left(\underset{k}{\sum }\alpha_{r}^{k}A^{k}\right), \end{array}$$ (8)

where $\alpha_{r}^{k}$ represents the importance weight for the *k*-th feature map in determining the final scalar value. The ReLU activation ensures that only features with a positive influence on the concentration estimate are visualized, effectively filtering out irrelevant background noise.

In this study, Grad-CAM was applied to the final convolutional layer of the Res-Reg backbone, as this layer provides the optimal balance between high-level chemical semantics and fine-grained spatial details. To generate the final visual explanations, the computed localization maps were upsampled via bilinear interpolation to match the original input image dimensions and overlaid as heatmaps. Because Grad-CAM provides a linear approximation of the model's behavior using first-order gradients (requiring only a single forward and partial backward pass), it is highly computationally efficient. This qualitative evaluation allows us to verify the model's decision-making process, ensuring that predictions are driven by chemically relevant optical signatures rather than potential biases or environmental artifacts.

### 2.6 Evaluation metrics

This study employed three evaluation metrics: Mean Absolute Error (MAE), also known as L1-loss, Mean Squared Error (MSE), and Geometric Mean (GM) error. MAE emphasizes the absolute value of errors, while MSE amplifies the impact of larger errors, ensuring the model adjusts more sensitively to significant discrepancies. These metrics provide a comprehensive assessment of the model's regression performance. Specifically, MAE (L1-loss) and MSE are defined by Equation 9 and Equation 10, respectively:


$$\begin{array}{l} MAE=\frac{1}{n}\sum_{i=1}^{n}|y_{i}-{ŷ}_{i}|, \end{array}$$ (9)

$$\begin{array}{l} MSE=\frac{1}{n}\sum_{i=1}^{n}{\left(y_{i}-{ŷ}_{i}\right)}^{2}, \end{array}$$ (10)

where *n* represents the number of samples in the image dataset, *y*_*i*_ denotes the true values, and ŷ_*i*_ represents the predicted results.

In addition to the commonly used loss functions, MAE and MSE, we also employed the GM, which is defined as Equation 11:


$$GM=\sqrt[{n}]{\prod_{i=1}^{n}\left|y_{i}-{\hat{y}}_{i}\right|}$$ (11)

By incorporating the GM, we achieve better prediction fairness, enabling a more balanced evaluation of the model's performance across different ranges of data. This approach helps to mitigate the bias that may arise from relying on a single error metric, ultimately improving the model's overall performance across various data types.

### 2.7 Implementation details

All models were implemented using PyTorch 2.1.1, with the CNN model trained on an NVIDIA 5070 Ti GPU. Additionally, the programming language used is Python 3.10.13, and the required packages include tensorboard-logger, numpy, pandas, scipy, among others. The workflow for model training is outlined as follows:

1. The image data was resized to 224 × 224 and normalized. Additionally, random cropping and random horizontal flipping were applied to the training set for data augmentation.
2. The label dataset was normalized to facilitate more efficient training and faster convergence. The original label dataset was also used for training to compare the results.
3. The composition and number of images in the dataset used in this study are provided in Table 1. The validation set was utilized exclusively for monitoring the training progress, optimizing hyperparameters, and implementing the early stopping strategy to prevent overfitting. Performance metrics are derived solely from the independent test set to ensure an unbiased evaluation of the model's generalization ability.
4. The proposed network framework is optimized and compiled employing the Adam optimizer. Adam adaptively adjusts the learning rate for each parameter by computing the first-order momentum (mean) and second-order momentum (variance) of the gradients. It has proven to be highly effective in handling issues such as sparse gradients, noisy gradients, and non-stationary objective functions.
5. In the hyperparameter settings, the initial learning rate is set to 0.001, the optimizer momentum is set to 0.9, and the weight decay for the optimizer is set to 0.0001.
6. Label Distribution Smoothing (LDS) and Feature Distribution Smoothing (FDS) are applied to address the issue of data imbalance.
7. The number of training epochs is set to 90, with a batch-size of 4.
8. The trained model is evaluated on the test set, and the final performance is assessed by calculating MAE, MSE, and GM.

**Table 1:** Composition and image count of the dataset.

Dataset	Train	Validation	Test	Total
original dataset	1,819	387	387	2,593
augmented dataset	7,276	1,548	1,548	10,372

The training hyperparameters are listed in Table 2.

**Table 2:** Training hyperparameters of the proposed model.

Epoch	Batch size	Optimizer	Learning rate	Momentum	Weight decay
90	4	adam	0.001	0.9	0.0001

## 3 Results and discussion

The existing original dataset and the augmented dataset constructed are used to test the effectiveness of this approach. To evaluate the performance of the proposed network architecture, different regression network models (VGG16, VGG19, ResNet18, ResNet34, ResNeXt, InceptionV3, EfficientNetB5) are trained on the training set. The models' performance is then evaluated on a test set consisting of 1,548 test images. The comparative results of the model performances are presented in Table 3.

**Table 3:** Performance of models on different datasets.

No.	Model	Original dataset					Enhanced Dataset				
		Epoch	Train loss	MSE	MAE	GM	Epoch	Train loss	MSE	MAE	GM
1	VGG16	54	3.98e0	3.97e0	1.71e0	2.61e0	33	3.98e0	3.97e0	1.71e0	2.61e0
2	VGG19	82	3.98e0	3.97e0	1.71e0	2.61e0	10	3.99e0	3.98e0	1.72e0	2.62e0
3	ResNet18	86	1.60e-3	3.00e-3	2.43e-2	8.55e-3	84	4.69e-4	1.67e-3	1.43e-2	4.88e-3
4	ResNet34	83	2.30e-3	1.59e-3	2.69e-2	6.55e-3	74	5.65e-4	5.76e-4	1.43e-2	2.87e-3
5	ResNet50	86	6.70e-3	1.13e-2	7.33e-2	2.87e-2	78	6.75e-4	2.22e-3	1.44e-2	5.66e-3
6	ResNeXt	81	4.00e-3	4.26e-3	4.39e-2	1.37e-2	83	6.47e-4	1.78e-3	1.62e-2	5.38e-3
7	InceptionV3	84	4.11e-2	8.62e-3	6.69e-2	2.40e-2	77	10.4e-2	8.05e-3	2.22e-2	1.34e-2
8	EfficientNetB5	82	1.20e-3	1.48e-3	2.09e-2	5.56e-3	80	8.15e-4	9.82e-5	7.23e-3	8.43e-4
9	**Res-Reg**	86	2.16e-2	1.20e-3	2.14e-2	9.90e-3	89	2.80e-3	3.00e-4	2.70e-3	2.00e-4

### 3.1 Evaluation of data augmentation method

**Figure 9:**
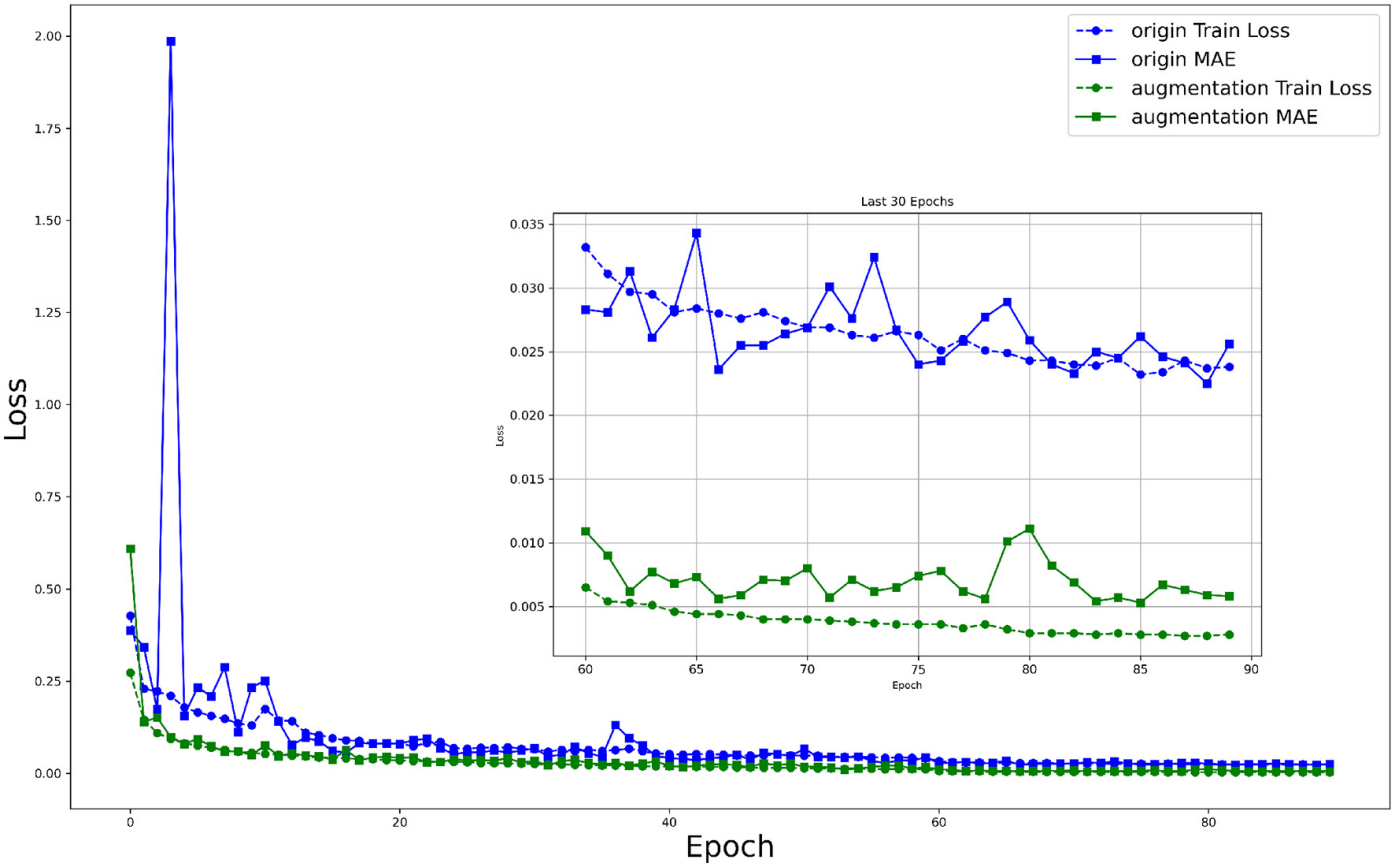
Training and testing loss curves (MAE) comparing the performance between the original dataset and the Gaussian-augmented dataset.

The original dataset (2,593 samples) and the augmented dataset (10,372 samples) were each used to train and test the same CNN model. The results are shown in Figure 9.

The trend analysis highlights the impact of data augmentation on model performance. For training error, the loss on the original dataset decreases gradually with epochs, indicating limited optimization and model fitting. In contrast, the augmented dataset demonstrates a steeper and more consistent reduction in training loss, suggesting that the inclusion of diverse samples improves learning and data fitting. For test error, the original dataset exhibits higher test loss, particularly in early stages, with minimal improvement in MAE, reflecting potential overfitting and poor generalization. In comparison, the augmented dataset achieves consistently lower test loss throughout training, indicating enhanced generalization and improved performance on unseen data.

Over the final 30 epochs, the training loss on the augmented dataset stabilizes, demonstrating a smoother convergence and more robust training process. Conversely, the training loss on the original dataset shows notable fluctuations, likely caused by insufficient data diversity, leading to optimization challenges and local minima. Similarly, for test error, the augmented dataset exhibits a smoother and more stable loss curve with lower values, highlighting its improved generalization capability. In contrast, the test loss for the original dataset is higher and more erratic, indicating unstable performance due to limited training data diversity and difficulty in adapting to test data distributions.

The comparative analysis clearly underscores the significant benefits of data augmentation. Models trained on the augmented dataset converge faster, exhibit lower training loss, and achieve substantially better MAE on the test set, reflecting stronger generalization and reduced overfitting. These findings confirm that data augmentation effectively enhances model robustness and performance, particularly in complex scenarios requiring better adaptability to diverse data distributions.

### 3.2 Evaluation of different network models

**Figure 10:**
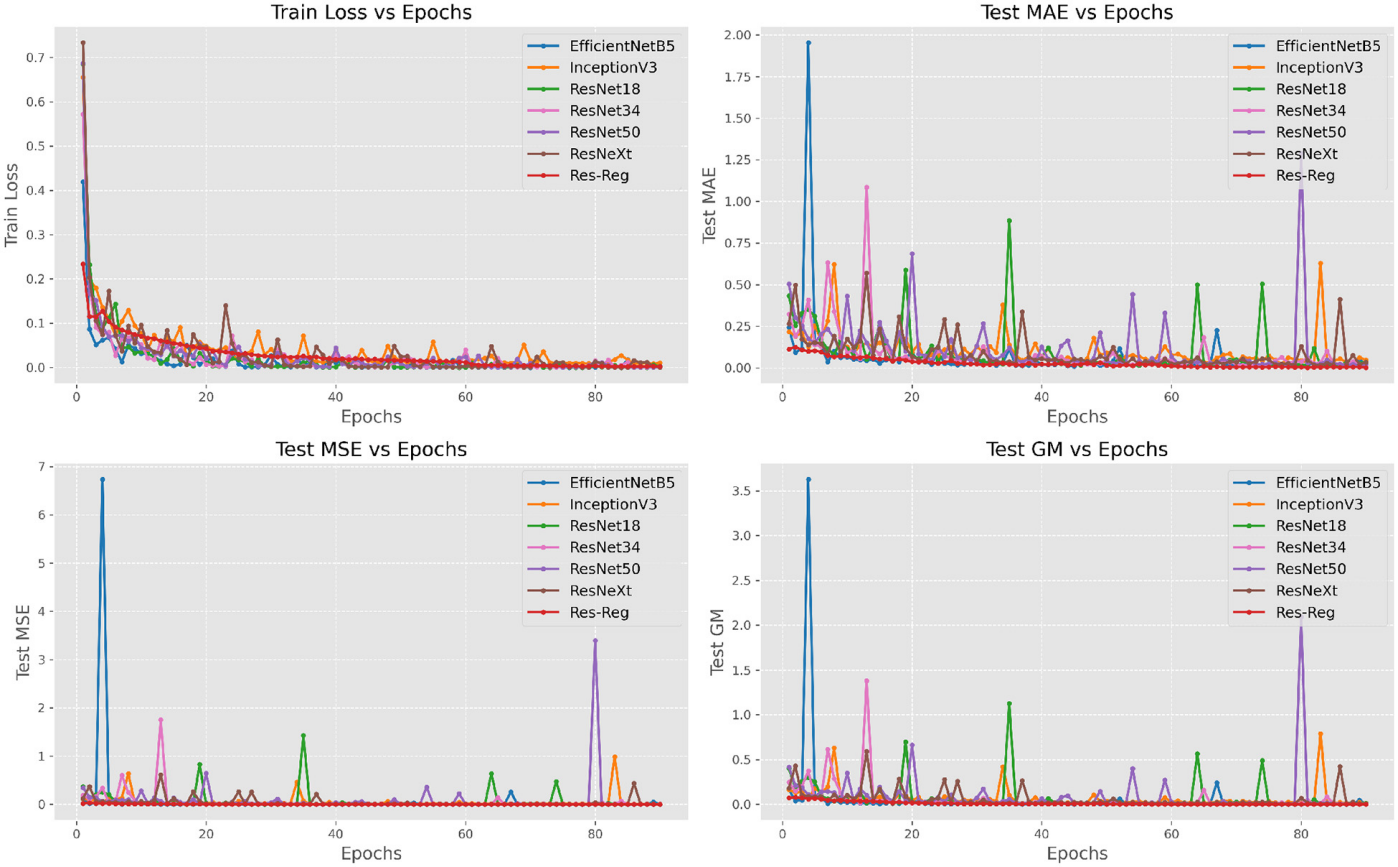
Quantitative performance comparison of Res-Reg against benchmark CNN architectures across 90 training epochs.

The objective of this study is to predict the concentration of formaldehyde solutions using an image dataset, leveraging the relationship between the optical properties of the images and the formaldehyde content. The model needs to effectively extract subtle feature information from the images. Since the optical characteristics of formaldehyde solutions in images may be affected by noise or blurring, Gaussian blur data augmentation is applied to enhance the model's robustness, enabling better performance when dealing with potential image blur or noise in real-world scenarios. To evaluate the performance of the proposed network architecture, a diverse suite of representative regression models–including VGG16, VGG19, ResNet18, ResNet34, ResNet50, ResNeXt, Inception V3 and EfficientNetB5–was selected for training. These benchmarks were specifically chosen to systematically evaluate the impact of increasing network depth, the effectiveness of residual connections, the advantages of multi-path feature extraction, and the performance of computationally efficient scaling architectures on the precision of formaldehyde quantification. The performance of each model was subsequently evaluated on an independent test set consisting of 1,548 images. The results are shown in Table 3. A selection of models with good performance and representative characteristics were chosen, and their loss during the training process is illustrated in the form of graphs, as shown in Figure 10.

In this study, we evaluated the performance of various CNN models on the formaldehyde high-throughput prediction dataset. The models were trained and tested using MAE as the loss function, and their performance was assessed based on metrics such as MAE, MSE, and GM. The custom-built Res-Reg demonstrated outstanding performance on the augmented dataset, achieving the lowest test MAE (0.0027) and GM (0.0002). These results highlight the model's precision in minimizing prediction errors and its robustness in handling diverse data distributions. Additionally, the training loss (0.0028) and test MSE (0.0003) confirm the model's ability to generalize effectively while maintaining stability across both training and testing phases. The performance of Res-Reg can be attributed to its architecture, which is well-optimized for extracting subtle features related to the optical properties of formaldehyde solutions. EfficientNetB5 achieved the lowest training loss (0.0008), indicating its effectiveness in capturing patterns during training. However, its higher test MAE (0.0072) and GM (0.0008) suggest potential overfitting or sensitivity to augmented features. While EfficientNetB5 excels in training efficiency, its ability to generalize to unseen data is limited, likely due to the complexity of its architecture being less tailored to the specific nuances of the formaldehyde dataset. InceptionV3 showed competitive performance in terms of test MSE (0.0081), demonstrating reasonable overall fit. However, its test MAE (0.0222) and GM (0.0134) were significantly higher than those of Res-Reg. This discrepancy suggests that InceptionV3 struggles with precise predictions, possibly due to its architectural complexity and reduced capacity to handle the data augmentation effects effectively. Both ResNet18 and ResNet34 exhibited stable performance, with ResNet34 slightly outperforming ResNet18 in GM (0.0029 vs. 0.0049). However, neither model matched the performance of Res-Reg. This indicates that while these residual networks can handle the dataset moderately well, deeper residual structures, such as Res-Reg, are more effective in capturing the fine-grained features required for high-throughput predictions.

The comparative analysis underscores the superiority of Res-Reg in formaldehyde high-throughput prediction tasks. Its balanced performance across all metrics demonstrates its ability to effectively learn and generalize from the augmented dataset. While other models, such as EfficientNetB5 and InceptionV3, showed strengths in specific aspects like training efficiency and overall fit, their performance on test data was less consistent, indicating challenges in generalization. These findings validate the design and implementation of Res-Reg as a reliable and robust model for this specific regression task.

### 3.3 Analysis of prediction results

**Figure 11:**
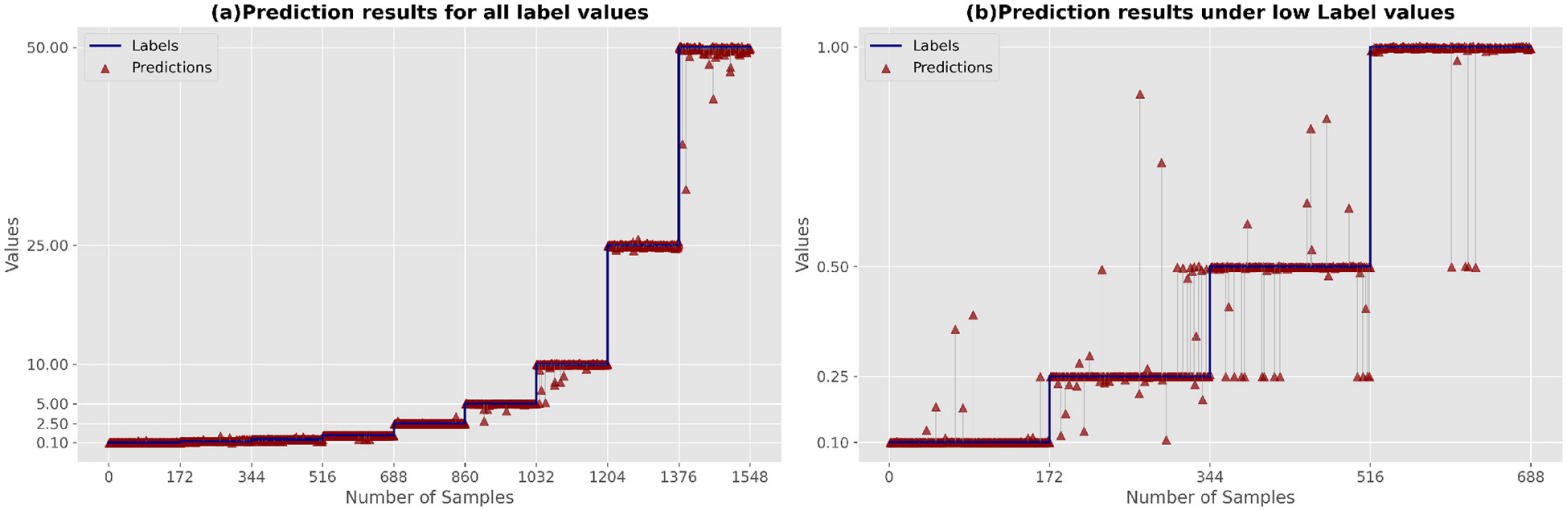
Prediction vs. ground-truth scatter plots: **(a)** Overall concentration spectrum; **(b)** Results in the low-concentration interval.

In this study, the trained regression network model was used to predict the values from image samples. The prediction results are shown in Figure 11, with detailed prediction errors presented in Table 4.

**Table 4:** Loss in the overall and low label value intervals.

Section	MSE	MAE	GM
Total images	0.0004	0.0045	0.0004
Images with low label	0.0009	0.0078	0.0002

**Figure 12:**
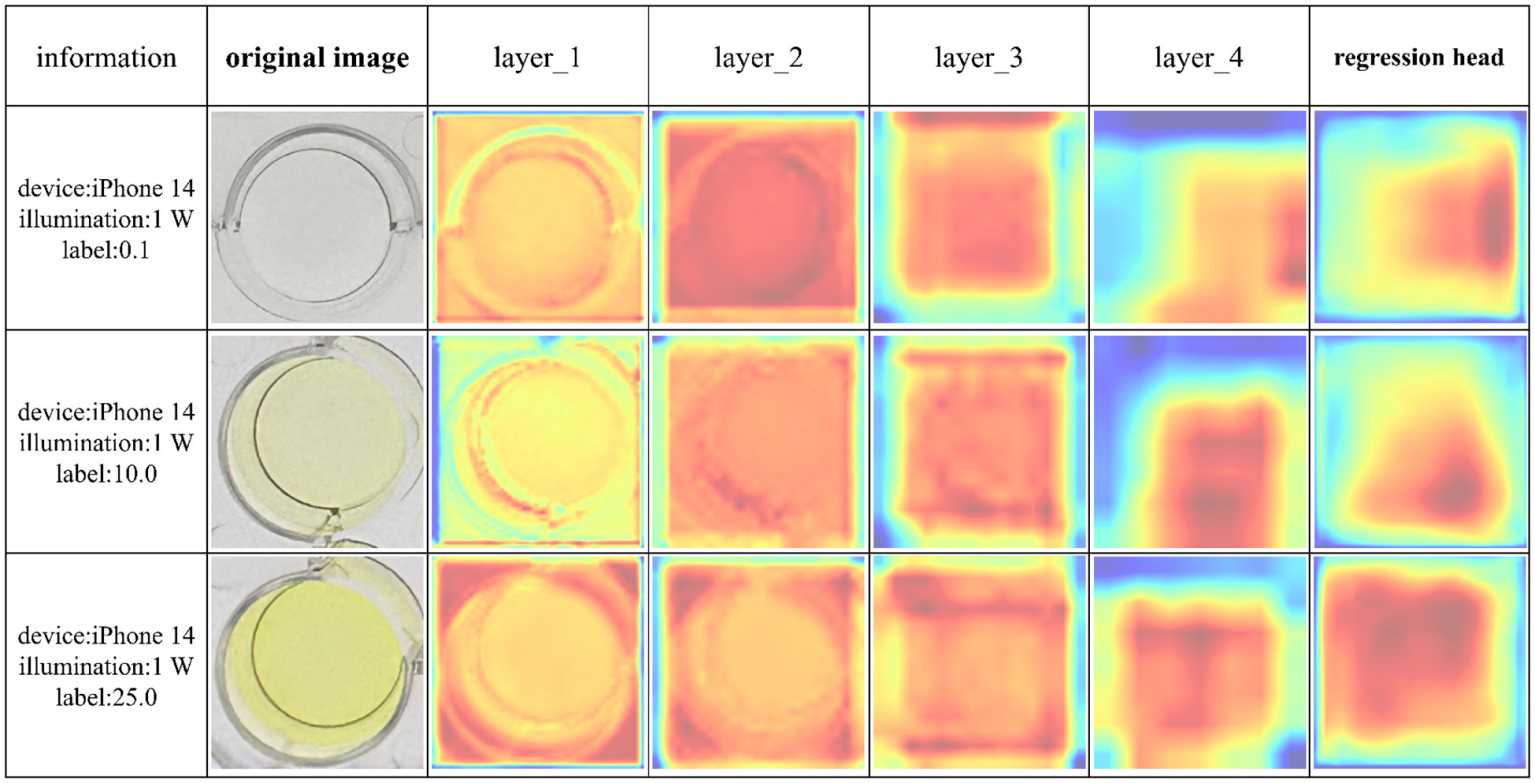
Grad-CAM visualization heatmaps across multiple network layers for samples at low, medium, and high formaldehyde concentrations.

The experimental results demonstrate that the model exhibits high prediction accuracy across the entire dataset (as shown in Figure 11a), with test errors such as MSE (0.0004), MAE (0.0045), and GM (0.0004) remaining at consistently low levels. This indicates that the model effectively captures the characteristics of formaldehyde solutions and provides accurate predictions for most samples. As shown in the figures, the model performs exceptionally well in the high label value range, with predicted values almost perfectly aligned with true values. This highlights the model's superior generalization ability in high-concentration formaldehyde samples, making it highly applicable to real-world high-throughput analysis. In the low label value range, the model continues to perform robustly. Figure 11b illustrates the model's predictions in the lower concentration range. While some samples exhibit slight deviations from true values, the overall prediction error remains minimal, providing valuable insights. Error analysis for this range reveals that MSE (0.0009) and GM (0.0002), though slightly higher than the overall error, are still within an extremely small range. This underscores the model's capability to handle low-concentration samples effectively.

To further confirm the methodological concordance between the Res-Reg framework and the conventional gold standard (UV-Vis spectrophotometry), correlation and linear regression analyses were performed (Table 5). The results indicate an exceptionally high Pearson correlation (*R* = 0.9992) and coefficient of determination (*R*^2^ = 0.9984), demonstrating that 99.84% of the variance is successfully captured by the model. Furthermore, the regression slope (*a* = 0.9919) and intercept (*b* = 0.0064) are near-ideal, confirming a stable and accurate linear mapping across the entire concentration spectrum with high statistical significance (*p* < 0.001). This high degree of correlation underscores that the proposed model provides results statistically equivalent to traditional laboratory instrumentation.

**Table 5:** Correlation statistics between Res-Reg predictions and the UV-Vis standard.

Category	Parameter	Value
**Correlation**	*R*	0.9992
	*R* ^2^	0.9984
**Regression**	Slope (*a*)	0.9919
	Intercept (*b*)	0.0064
	*p*-value	< 0.001

*R*: Pearson correlation coefficient; *R*^2^: Coefficient of determination. The regression equation is defined as *y* = *ax*+*b*.

To rigorously address potential concerns regarding the model's reliability in these high-sensitivity intervals, a statistical comparison was performed to determine if the slight variation in absolute prediction errors between the low-label and high-label regions was statistically significant. The dataset was partitioned into a low-label interval ( ≤ 1.0mg/L, *n* = 688) and a high-label interval (>1.0mg/L, *n* = 860). A Welch's one-sided t-test was conducted with the alternative hypothesis that errors in the low-concentration “gray zone” are significantly higher than those in high-concentration samples. The comprehensive statistical results are summarized in Table 6.

**Table 6:** Statistical comparison of prediction errors between low-label and high-label concentration intervals.

Metric	Interval	*n*	Mean	SD	*t*-statistic	*p*-value
**MSE**	Low-label ( ≤ 1.0)	688	0.0075	0.0588	-1.859	0.968
	High-label (>1.0)	860	0.7971	12.4593		
**MAE**	Low-label ( ≤ 1.0)	688	0.0205	0.0840	-5.010	0.999
	High-label (>1.0)	860	0.1712	0.8768		

Welch's one-sided t-test was performed (*H*_1_:Error_low_>Error_high_). *p*≥0.05 indicates that prediction errors in the low-concentration “gray zone” are not significantly higher than those in high-concentration samples.

As detailed in Table 6, the analysis yielded *p*-values that are exceptionally close to 1.0 (*p* = 0.968 for MSE and *p* = 0.999 for MAE), falling well above the standard 0.05 significance threshold. Consequently, we fail to reject the null hypothesis. The negative *t*-statistics further reveal a critical insight: the mean prediction errors in the challenging low-concentration interval (e.g., Mean MAE = 0.0205) are, in fact, significantly lower in absolute magnitude compared to the high-concentration interval (Mean MAE = 0.1712). The larger standard deviation (SD) observed in the high-label group is a common analytical phenomenon where absolute residuals scale proportionately with higher analyte concentrations. Ultimately, this statistical evidence definitively confirms that the Res-Reg framework experiences no significant degradation in performance at near-detection limits. It maintains consistent, laboratory-grade precision across the entire dynamic range, successfully mitigating the optical interferences typically associated with complex food matrices.

To evaluate the cross-device robustness of the proposed framework, the predictive performance was systematically compared across four heterogeneous smartphone models (Table 7). Despite the inherent variations in CMOS sensor sensitivities and built-in image processing algorithms among the devices, the Res-Reg model maintained exceptional precision. Notably, the *R*^2^ values for all models exceeded 0.994, with the iPhone 14 achieving a near-perfect correlation of 0.9999. The slight fluctuations in MAE (ranging from 0.0504 to 0.1722 mg/L) across brands like Apple, OPPO, and Redmi underscore the model's ability to prioritize intrinsic chemical chromogenic patterns over hardware-specific chromatic renderings. This consistency validates the framework's 'onsite scalability,' confirming that reliable food safety monitoring can be achieved regardless of the specific smartphone hardware utilized by the end-user.

**Table 7:** Quantitative performance comparison of the Res-Reg framework across heterogeneous smartphone devices.

Smartphone model	Sample size (*n*)	*R* ^2^	MAE (mg/L)	RMSE (mg/L)
iPhone 11	372	0.9993	0.1017	0.4253
iPhone 14	398	0.9999	0.0504	0.1633
OPPO Reno5	385	0.9945	0.1722	1.1538
Redmi Note 11t Pro	393	0.9994	0.0838	0.3654
**Overall**	**1548**	**0.9984**	**0.1015**	**0.6444**

*R*^2^: Coefficient of determination; MAE, Mean Absolute Error; RMSE, Root Mean Square Error. All predictions were performed on the standardized test set using the same Res-Reg parameters. Bold values indicate the overall performance.

The robustness of the Res-Reg framework to lighting variations was validated by comparing its performance under two distinct intensities: 1W and 5W (Table 8). Although a higher light intensity (5W) yielded slightly superior precision (*R*^2^ = 0.9996, MAE = 0.0685mg/L), the performance under the lower 1W intensity remained exceptionally stable with an *R*^2^ of 0.9972. This negligible difference indicates that the deep residual architecture has successfully learned to decouple the intrinsic chemical chromogenic features from absolute photon flux variations. Such 'intensity-invariance' precludes the need for complex manual lighting calibration or additional intensity-based data inputs, further simplifying the operational requirements for on-site food safety monitoring.

**Table 8:** Robustness of Res-Reg predictions under different light intensities (1W and 5W).

Light Intensity	Sample Size (*n*)	*R* ^2^	MAE (mg/L)	RMSE (mg/L)
1W	794	0.9972	0.1328	0.8435
5W	754	0.9996	0.0685	0.3215
**Combined**	**1548**	**0.9984**	**0.1015**	**0.6444**

*R*^2^: Coefficient of determination; MAE, Mean Absolute Error; RMSE, Root Mean Square Error. Bold values indicate the overall performance.

To assess the feasibility of Res-Reg for real-time on-site inspections, we evaluated its inference latency. On a high-performance workstation (NVIDIA GeForce RTX 5070 Ti), the model processed the augmented test set of 10,372 images in a total of 2.498 seconds, achieving an average inference time of 1.61 ms per sample. While these benchmarks were obtained on desktop-grade hardware, the millisecond-level latency highlights the framework's exceptional computational efficiency. Such a low inference overhead provides significant 'computational headroom' for migration to mobile platforms. Even considering the lower clock speeds and power constraints of mobile SoC (System on Chip) architectures, the current efficiency suggests that Res-Reg could achieve near-instantaneous results on consumer smartphones. Furthermore, in network-accessible scenarios, an API-based architecture can be utilized to offload intensive tasks to high-performance remote servers. This cloud-assisted inference paradigm would allow mobile terminals to leverage desktop-grade processing speeds regardless of local hardware limitations. Future optimization through model quantization and edge-computing frameworks (e.g., CoreML or TensorRT-Mobile) will further solidify its potential for both offline and online, zero-latency food safety monitoring.

The challenges and limitations of this experiment are as follows. First, the Hantzsch reaction is sensitive to pH and incubation temperature, which govern chromogenic kinetics and color intensity. Second, optical interferences from container transparency, light scattering at interfaces, and substrate turbidity (e.g., suspended proteins or lipids) can introduce systematic offsets and attenuate the signal-to-noise ratio. Moreover, the method is not applicable to compounds lacking reactive carbonyl groups, and potential interference from structural analogs or substances with native absorption at 415 nm remains a challenge for specificity in complex matrices. Third, although tested on four smartphones, the inherent diversity of CMOS sensors and proprietary image algorithms necessitates standardized calibration for universal hardware scalability. Fourth, reliance on high-performance GPUs (e.g., NVIDIA RTX 5070 Ti) limits offline deployment in resource-constrained environments. Future work will explore model quantization and cloud-edge collaboration to enhance the portability and cross-platform adaptability of the Res-Reg framework. Finally, it is important to acknowledge that while the Res-Reg framework was rigorously evaluated using simulated aquatic product soaking solutions designed to replicate the turbidity and complex biochemical backgrounds of real seafood matrices, the current study did not include a large-scale validation using diverse fresh aquatic samples directly obtained from markets. To ensure standardized sample preparation and the initial convergence of the model, validation was focused on these high-fidelity simulated environments. Nevertheless, this represents a limitation of the present work, as authentic samples from different species (e.g., various fish or shrimp) may introduce additional biochemical variance. Consequently, future research will prioritize overcoming these infrastructure barriers to integrate real-world samples from diverse aquatic species, further refining the model's robustness in practical, on-site food safety surveillance scenarios.

### 3.4 Visualization of the regression network

This study employs Grad-CAM to visualize and analyze the decision-making process of the proposed Res-Reg model for formaldehyde concentration prediction in image regression tasks. By highlighting the spatial regions of the input images that exert the most influence on the predicted concentration, Grad-CAM allows us to evaluate whether the model's attention correctly aligns with chemically relevant features. The analysis spans feature visualizations from the backbone network (Layers 1-4) to the final regression head, providing insights into both the hierarchical feature evolution and the model's stability across varying concentration levels.

As shown in Figure 12, the Grad-CAM heatmaps reveal that the Res-Reg model progressively shifts its focus from low-level local features in shallow layers to high-level global semantic features in deeper layers. In Layer 1, the model primarily attends to structural edges and local textures, which help define the physical boundaries of the reaction well. Through Layers 2 and 3, attention expands to larger regions, beginning to identify color gradients and broader structural patterns within the solution. By Layer 4, the attention narrows specifically to the core regions of the solution where significant variations in formaldehyde concentration (and thus, color intensity) occur, reflecting the successful extraction of abstract, chemistry-related features.

The regression head, a critical component of the Res-Reg framework, maps these high-dimensional features extracted by the convolutional backbone to the final continuous scalar output. Its architecture utilizes upsampling layers to enlarge feature maps and 1 × 1 convolutions to reduce channel dimensions, ultimately generating a density map that is processed by a fully connected layer. The Grad-CAM visualizations demonstrate that the regression head effectively synthesizes features from earlier layers, concentrating firmly on regions exhibiting the most significant chromogenic shifts.

Crucially, to verify the stability and robustness of the Res-Reg framework, we analyzed the Grad-CAM heatmaps across different target classes in the regression context: low (0.1mg/L), medium (10.0mg/L), and high (25.0mg/L) concentration intervals (as depicted in the rows of Figure 12). In low concentration (0.1mg/L), the liquid appears nearly transparent to the naked eye. However, the model successfully captures subtle variations in optical texture and localized chromophore accumulation, maintaining its focus firmly within the well. In medium to high Concentration (10.0mg/L and 25.0mg/L), as the color deepens to a saturated yellow, the attention profile becomes more concentrated and spatially uniform, precisely aligning with the strong signal of the 3,5-diacetyl-1,4-dihydrolutidine product in the center of the solution.

Despite the drastic shifts in overall color intensity and clarity across the samples, the model's attention remains consistently anchored within the reaction well's center. This cross-concentration stability confirms that the model does not merely memorize background artifacts or environmental noise. Instead, this progressive shift in attention—from local edges to global chemical features—underscores the model's capacity to adaptively learn and base its precise regression predictions on authentic, chemically relevant optical signals.

While Grad-CAM visualizations confirm that Res-Reg effectively prioritizes the chromogenic regions, a critical consideration in real-world deployment is the mitigation of potential biases, such as false positives. In complex aquatic matrices, non-target features—including microscopic debris, air bubbles, or subtle unevenness in illumination—could theoretically be misinterpreted by the model as indicative of higher formaldehyde concentrations.

To address these biases, the Res-Reg framework integrates two primary safeguards. First, the inclusion of multi-scale feature aggregation in the regression head ensures that the model evaluates both local color intensity and global spatial distribution, preventing it from over-focusing on isolated artifacts. Second, by incorporating specific data augmentation strategies, such as Gaussian noise and blur kernels during training, the network is forced to learn robust representation patterns that distinguish intrinsic chemical signals from environmental noise. These measures significantly enhance the framework's discriminative reliability, ensuring that the high-throughput predictions remain grounded in the authentic Hantzsch reaction signatures rather than matrix-induced artifacts.

## 4 Conclusion

This study successfully developed and validated Res-Reg, a novel deep-regression framework designed to bridge the gap between microscopic chemical sensing and high-throughput food safety monitoring. By synergizing the robust chemical kinetics of the Hantzsch chromogenic reaction with advanced residual feature aggregation, we have demonstrated a streamlined analytical ecosystem capable of quantifying formaldehyde (FA) concentrations with laboratory-grade precision. Unlike traditional qualitative or classification-based approaches, the Res-Reg system leverages a specialized regression head to achieve an ultra-accurate “image-to-value” mapping, effectively perceiving infinitesimal optical shifts in the chromophore 3,5-diacetyl-1,4-dihydrolutidine (DDL) that remain imperceptible to the human eye or conventional machine learning models.

The practical significance of this research is underscored by its superior performance in simulated food matrices, specifically aquatic product soaking solutions. These substrates, characterized by inherent turbidity and complex biochemical backgrounds, typically pose significant interference to optical sensing; however, the Res-Reg framework demonstrated exceptional robustness by capturing granular-level reaction features through microscopic imaging. Furthermore, the integration of a Gaussian blur-based data augmentation pipeline ensures the system's reliability against the optical degradations common in onsite industrial environments, such as focus instability and ambient lighting fluctuations. This capability to maintain high precision within low-concentration “gray zones” is critical for ensuring compliance with international food safety standards and protecting consumers from the chronic health risks associated with FA ingestion.

From a broader perspective, the Res-Reg framework represents a significant step toward the realization of Food Industry 4.0, providing a versatile and scalable tool for the digital transformation of food quality control. The modular nature of the architecture allows for its potential adaptation to other critical food contaminants or nutritional markers across diverse food matrices without the need for centralized laboratory infrastructure. By eliminating the bottlenecks of labor-intensive sample pre-treatment and expensive instrumentation, this technology offers a sustainable and cost-effective solution for proactive food safety surveillance. Future work will focus on integrating this deep-regression model into portable smart devices, thereby empowering stakeholders across the global food supply chain with real-time, data-driven insights to safeguard nutritional integrity and public health.

## Data Availability

The datasets presented in this study can be found in online repositories. The names of the repository/repositories and accession number(s) can be found in the article/supplementary material.
